# Supplementary material for: Evolutionary plasticity and functional repurposing of the essential metabolic enzyme MoeA
Source: Commun Biol. 2025 Jan 14;8:49. doi: 10.1038/s42003-025-07476-3 (PMC11733289; doi:10.1038/s42003-025-07476-3)
Supplement: Supplementary file 8 — Description of Additional Supplementary Files [file 42003_2025_7476_MOESM8_ESM.docx]

Description of Additional Supplementary Files

**File name:** Supplementary Data 1

**Description:** Genbank protein ids of MoeA homologs identified in Archaea, Bacteria and Eukaryotes.

**File name:** Supplementary Data 2

**Description:** Genbank protein ids of ModA, MoeAPBP, WtpA and TupA homologs identified in the Archaea and Bacteria databases.

**File name:** Supplementary Data 3

**Description:** Genbank protein ids of MoeA-PBP and TupA homologs identified in all representative complete genomes of Bacteria.

**File name:** Supplementary Data 4

**Description:** Pairwise distances between the residues of interest in the MoeA structures used for the PCA analyses.

**File name:** Supplementary Data 5

**Description:** List of Genbank protein ids of the selected structures used for mapping the sequence conservation.

**File name:** Supplementary Data 6

**Description:** List of residues positions of the active site included in the distance calculation analysis, relative to E. coli Genbank protein sequence AIZ54672.1.
