# Supplementary material for: Evolutionary plasticity and functional repurposing of the essential metabolic enzyme MoeA
Source: Commun Biol. 2025 Jan 14;8:49. doi: 10.1038/s42003-025-07476-3 (PMC11733289; doi:10.1038/s42003-025-07476-3)
Supplement: Supplementary file 9 — nr-reporting-summary [file 42003_2025_7476_MOESM9_ESM.pdf]

nature portfolio

Corresponding author(s): Anne Marie WEHENKEL, Daniela MEGRIAN

Last updated by author(s): Dec 9, 2024

Reporting Summary

Nature Portfolio wishes to improve the reproducibility of the work that we publish. This form provides structure for consistency and transparency in reporting. For further information on Nature Portfolio policies, see our Editorial Policies and the Editorial Policy Checklist.

Statistics

For all statistical analyses, confirm that the following items are present in the figure legend, table legend, main text, or Methods section.

n/a	Confirmed	
☒	☐	The exact sample size (*n*) for each experimental group/condition, given as a discrete number and unit of measurement
☒	☐	A statement on whether measurements were taken from distinct samples or whether the same sample was measured repeatedly
☒	☐	The statistical test(s) used AND whether they are one- or two-sided<br>*Only common tests should be described solely by name; describe more complex techniques in the Methods section.*
☒	☐	A description of all covariates tested
☒	☐	A description of any assumptions or corrections, such as tests of normality and adjustment for multiple comparisons
☒	☐	A full description of the statistical parameters including central tendency (e.g. means) or other basic estimates (e.g. regression coefficient) AND variation (e.g. standard deviation) or associated estimates of uncertainty (e.g. confidence intervals)
☒	☐	For null hypothesis testing, the test statistic (e.g. *F*, *t*, *r*) with confidence intervals, effect sizes, degrees of freedom and *P* value noted<br>*Give P values as exact values whenever suitable.*
☒	☐	For Bayesian analysis, information on the choice of priors and Markov chain Monte Carlo settings
☒	☐	For hierarchical and complex designs, identification of the appropriate level for tests and full reporting of outcomes
☒	☐	Estimates of effect sizes (e.g. Cohen's *d*, Pearson's *r*), indicating how they were calculated

Our web collection on statistics for biologists contains articles on many of the points above.

Software and code

Policy information about availability of computer code

Data collection

No software was used for data collection.

Data analysis

- Homology searches:
HMMER-v3.3: hmmbuild to build hidden Markov models (HMM) profiles, and jackhmmer and hmmsearch for sequence similarity searches against databases using a sequence or an HMM profile, respectively.
Conserved Domain Database (CDD): online tool, version available between 2022 and 2024.

- Evolutionary analysis:
MAFFT v7.407: with the L-INS-I option for the alignments
trimAl v1.4.rev15: to remove the columns that contain less than 20% of gaps in the alignments (option -gt 0.2)
IQ-TREE v2.0.6: with the model LG+C60+F+I+G (+PMSF).

- Phylogeny visualisation and mapping:
iTOL: online tool, versions available between 2022 and 2024.

- Protein structure prediction:
Alphafold v2.3.0: using multimer model parameters.

- Protein structure visualisation:
ChimeraX v1.6: using the AL2CO software for calculating the sequence conservation.

- Distance calculation between protein residues:
Python Bio.PDB package v1.75: using module PDBParser.

For manuscripts utilizing custom algorithms or software that are central to the research but not yet described in published literature, software must be made available to editors and reviewers. We strongly encourage code deposition in a community repository (e.g. GitHub). See the Nature Portfolio guidelines for submitting code & software for further information.

Data

Policy information about availability of data

All manuscripts must include a data availability statement. This statement should provide the following information, where applicable:

- Accession codes, unique identifiers, or web links for publicly available datasets
- A description of any restrictions on data availability
- For clinical datasets or third party data, please ensure that the statement adheres to our policy

All data supporting the findings of this study are available within the paper and its Supplementary Figures and Supplementary Data. Source Data and can be found in https://doi.org/10.17632/phw4knbn8m.2. All other data are available from the corresponding author.

Research involving human participants, their data, or biological material

Policy information about studies with human participants or human data. See also policy information about sex, gender (identity/presentation), and sexual orientation and race, ethnicity and racism.

Reporting on sex and gender	NA
Reporting on race, ethnicity, or other socially relevant groupings	NA
Population characteristics	NA
Recruitment	NA
Ethics oversight	NA

Note that full information on the approval of the study protocol must also be provided in the manuscript.

Field-specific reporting

Please select the one below that is the best fit for your research. If you are not sure, read the appropriate sections before making your selection.

☐ Life sciences ☐ Behavioural & social sciences ☒ Ecological, evolutionary & environmental sciences

For a reference copy of the document with all sections, see nature.com/documents/nr-reporting-summary-flat.pdf

Ecological, evolutionary & environmental sciences study design

All studies must disclose on these points even when the disclosure is negative.

Study description	We looked for MoeA homologs in genomes representing all bacterial, archaeal and eukaryotic diversity. We identified conserved domains in each putative homolog, and we analysed the evolution and presence/absence pattern in the genomes. To do this, we reconstructed MoeA phylogenies based on the homologs identified in all domains of life.<br>We looked for ModA, WtpA and TupA homologs, and we explored the co-occurrence of these transporters and MoeA homologs fused to a PBP domain.<br>Finally, we predicted the structure of the dimeric form of representative MoeA homologs from all domains of life. We analysed the sequence conservation in the active site, and the conservation of the distances between the residues involved in the active site. Finally, we compared these attributes between the different MoeA groups defined based on the phylogenetic analyses.
Research sample	Four databases were used in our analyses:<br>- 81 bacterial genomes<br>- 122 archaeal genomes<br>- 129 eukaryotic genomes<br>- 171 fungal genomes
Sampling strategy	For Bacteria, we selected five taxa per phylum with cultured representatives. For Archaea, we assembled a database containing 122 genomes representing all major phyla, based on a previous taxonomic sampling, but excluding the genomes that are not annotated. For Eukaryotes, we selected five taxa per phylum (if available), from all eukaryotic annotated genomes. For Fungi, we included one representative of each fungal order with at least one annotated genome.

Data collection	All genomes were downloaded from the NCBI Genome database.
Timing and spatial scale	NA
Data exclusions	NA
Reproducibility	The reliability of the phylogenetic trees was measured by the bootstrap probability of interior branches of the tree. We used the ultrafast bootstrap approximation implemented in the IQ-TREE software with 1000 replicates.
Randomization	This kind of procedure does not apply to phylogenetic analysis
Blinding	This kind of procedure does not apply to phylogenetic analysis

Did the study involve field work? ☐ Yes ☒ No

Reporting for specific materials, systems and methods

We require information from authors about some types of materials, experimental systems and methods used in many studies. Here, indicate whether each material, system or method listed is relevant to your study. If you are not sure if a list item applies to your research, read the appropriate section before selecting a response.

Materials & experimental systems

n/a	Involved in the study
☒	☐ Antibodies
☒	☐ Eukaryotic cell lines
☒	☐ Palaeontology and archaeology
☒	☐ Animals and other organisms
☒	☐ Clinical data
☒	☐ Dual use research of concern
☒	☐ Plants

Methods

n/a	Involved in the study
☒	☐ ChIP-seq
☒	☐ Flow cytometry
☒	☐ MRI-based neuroimaging

Plants

Seed stocks	NA
Novel plant genotypes	NA
Authentication	NA
